# Supplementary material for: Development of Clinical Decision Models for the Prediction of Systemic Lupus Erythematosus and Sjogren’s Syndrome Overlap
Source: J Clin Med. 2023 Jan 9;12(2):535. doi: 10.3390/jcm12020535 (PMC9862529; doi:10.3390/jcm12020535)
Supplement: Supplementary file 1 [file jcm-12-00535-s001.zip › jcm-2126267-supplementary.pdf]

Supplementary Materials

Table S1 Patient demographic and clinical characteristics in discovery and validation cohorts

	SLE vs SLE-SS				SS vs SLE-SS			
**Variable**	**ALL (N = 290)**	**Discovery cohort (N = 203)**	**Validation cohort (N = 87)**	**P value**	**ALL (N = 266)**	**Discovery cohort (N = 187)**	**Validation cohort (N = 79)**	**P value**
**Demographic features**								
Female	266 (91.72%)	187 (92.12%)	79 (90.80%)	0.89	243 (91.35%)	171 (91.44%)	72 (91.14%)	1.00
Age at diagnosis (years)	36 [27-49]	36 [26.5-49]	37 [28-47.5]	0.33	48 [32.25-59.75]	48 [32-61]	48 [37-58]	0.97
Complicated with hypertension	95 (32.76%)	69 (33.99%)	26 (29.89%)	0.59	57 (21.43%)	38 (20.32%)	19 (24.05%)	0.61
Complicated with diabetes	13 (4.48%)	8 (3.94%)	5 (5.75%)	0.54	23 (8.65%)	16 (8.56%)	7 (8.86%)	1.00
**Clinical features**								
Fever	127 (43.79%)	85 (41.87%)	42 (48.28%)	0.38	63 (23.68%)	45 (24.06%)	18 (22.78%)	0.95
Alopecia	62 (21.38%)	44 (21.67%)	18 (20.69%)	0.98	27 (10.15%)	17 (9.09%)	10 (12.66%)	0.51
Asthenia	48 (16.55%)	26 (12.81%)	22 (25.29%)	0.01	54 (20.30%)	43 (22.99%)	11 (13.92%)	0.13
Dry mouth	76 (26.21%)	52 (25.62%)	24 (27.59%)	0.84	224 (84.21%)	156 (83.42%)	68 (86.08%)	0.72
Dry eye	46 (15.86%)	34 (16.75%)	12 (13.79%)	0.65	157 (59.02%)	109 (58.29%)	48 (60.76%)	0.81
Photosensitivity	71 (24.48%)	52 (25.62%)	19 (21.84%)	0.59	24 (9.02%)	19 (10.16%)	5 (6.33%)	0.45
Skin lesions	173 (59.66%)	122 (60.10%)	51 (58.62%)	0.92	59 (22.18%)	39 (20.86%)	20 (25.32%)	0.52
Oral ulcer	50 (17.24%)	37 (18.23%)	13 (14.94%)	0.61	39 (14.66%)	27 (14.44%)	12 (15.19%)	1.00
Raynaud phenomenon	57 (19.66%)	39 (19.21%)	18 (20.69%)	0.90	56 (21.05%)	36 (19.25%)	20 (25.32%)	0.35

Cavities	19 (6.55%)	13 (6.40%)	6 (6.90%)	1.00	49 (18.42%)	35 (18.72%)	14 (17.72%)	0.99
Epistaxis	17 (5.86%)	12 (5.91%)	5 (5.75%)	1.00	17 (6.39%)	11 (5.88%)	6 (7.59%)	0.81
Arthritis	167 (57.59%)	107 (52.71%)	60 (68.97%)	0.02	96 (36.09%)	65 (34.76%)	31 (39.24%)	0.58
Proteinuria	176 (60.69%)	127 (62.56%)	49 (56.32%)	0.39	87 (32.71%)	62 (33.16%)	25 (31.65%)	0.92
Hematuria	155 (53.45%)	109 (53.69%)	46 (52.87%)	1.00	75 (28.20%)	53 (28.34%)	22 (27.85%)	1.00
Interstitial lung disease	17 (5.86%)	11 (5.42%)	6 (6.90%)	0.83	39 (14.66%)	26 (13.90%)	13 (16.46%)	0.73
Pulmonary arterial hypertension	65 (22.41%)	41 (20.20%)	24 (27.59%)	0.22	47 (17.67%)	29 (15.51%)	18 (22.78%)	0.21
Vasculitis	21 (7.24%)	18 (8.87%)	3 (3.45%)	0.17	9 (3.38%)	7 (3.74%)	2 (2.53%)	1.00
**Laboratory findings**								
Anemia	151 (52.07%)	105 (51.72%)	46 (52.87%)	0.96	95 (35.71%)	69 (36.90%)	26 (32.91%)	0.63
Leukocytopenia	106 (36.55%)	75 (36.95%)	31 (35.63%)	0.94	94 (35.34%)	63 (33.69%)	31 (39.24%)	0.47
Thrombocytopenia	68 (23.45%)	45 (22.17%)	23 (26.44%)	0.53	58 (21.80%)	40 (21.39%)	18 (22.78%)	0.93
Hypoalbuminemia	152 (52.41%)	109 (53.69%)	43 (49.43%)	0.59	70 (26.32%)	51 (27.27%)	19 (24.05%)	0.69
Increased blood urea nitrogen	81 (27.93%)	60 (29.56%)	21 (24.14%)	0.42	23 (8.65%)	18 (9.63%)	5 (6.33%)	0.53
Increased serum creatinine	43 (14.83%)	36 (17.73%)	7 (8.05%)	0.05	11 (4.14%)	10 (5.35%)	1 (1.27%)	0.18
Increased erythrocyte sedimentation rate	194 (66.90%)	131 (64.53%)	63 (72.41%)	0.24	153 (57.52%)	113 (60.43%)	40 (50.63%)	0.18
Increased C-reactive protein	111 (38.28%)	71 (34.98%)	40 (45.98%)	0.10	77 (28.95%)	54 (28.88%)	23 (29.11%)	1.00
Hypocomplementemia	205 (70.69%)	141 (69.46%)	64 (73.56%)	0.57	183 (68.80%)	131 (70.05%)	52 (65.82%)	0.59
ANA positive	156 (53.79%)	112 (55.17%)	44 (50.57%)	0.55	123 (46.24%)	87 (46.52%)	36 (45.57%)	0.99
Anti-dsDNA positive	169 (58.28%)	117 (57.64%)	52 (59.77%)	0.84	67 (25.19%)	44 (23.53%)	23 (29.11%)	0.42
Anti-SSA positive	187 (64.48%)	130 (64.04%)	57 (65.52%)	0.92	197 (74.06%)	137 (73.26%)	60 (75.95%)	0.76
Anti-Ro52 positive	126 (43.45%)	88 (43.35%)	38 (43.68%)	1.00	152 (57.14%)	108 (57.75%)	44 (55.70%)	0.86

Anti- SSB positive	65 (22.41%)	48 (23.65%)	17 (19.54%)	0.54	96 (36.09%)	64 (34.22%)	32 (40.51%)	0.40
Anti-Sm positive	97 (33.45%)	70 (34.48%)	27 (31.03%)	0.66	33 (12.41%)	20 (10.70%)	13 (16.46%)	0.27
Anti-RNP positive	69 (23.79%)	47 (23.15%)	22 (25.29%)	0.81	40 (15.04%)	24 (12.83%)	16 (20.25%)	0.17
Anti-cardiolipin positive	22/217 (10.14%)	16/151 (10.60%)	6/66 (9.09%)	0.93	17/193 (8.81%)	12/141 (8.51%)	5/52 (9.62%)	0.78
RF positive	49 (16.90%)	32 (15.76%)	17 (19.54%)	0.54	89 (33.46%)	61 (32.62%)	28 (35.44%)	0.76
IgG elevation	83 (28.62%)	52 (25.62%)	31 (35.63%)	0.11	90 (33.83%)	63 (33.69%)	27 (34.18%)	1.00

Table S2. Clinical characteristics of SLE and SLE-SS in discovery and validation cohorts.

	Discovery cohort			Validation cohort		
**Characteristics**	**SLE-SS (N = 53)**	**SLE (N = 150)**	**P value**	**SLE-SS (N = 26)**	**SLE (N = 61)**	**P value**
**Demographic features**						
Female	51 (96.23%)	136 (90.67%)	0.25	24 (92.31%)	55 (90.16%)	1.00
Age at diagnosis (years)	33.5 [26-47.75]	36 [27-51]	0.31	43.5 [31.5-50]	33 [27-45]	0.04
Complicated with hypertension	9 (16.98%)	60 (40.00%)	0.00	6 (23.08%)	20 (32.79%)	0.52
Complicated with diabetes	2 (3.77%)	6 (4.00%)	1.00	1 (3.85%)	4 (6.56%)	1.00
**Clinical features**					(%)	
Fever	26 (49.06%)	59 (39.33%)	0.28	8 (30.77%)	34 (55.74%)	0.06
Alopecia	8 (15.09%)	36 (24.00%)	0.25	6 (23.08%)	12 (19.67%)	0.94
Asthenia	10 (18.87%)	16 (10.67%)	0.20	8 (30.77%)	14 (22.95%)	0.62
Dry mouth	34 (64.15%)	18 (12.00%)	0.00	18 (69.23%)	6 (9.84%)	0.00
Dry eye	25 (47.17%)	9 (6.00%)	0.00	10 (38.46%)	2 (3.28%)	0.00
Photosensitivity	15 (28.30%)	37 (24.67%)	0.74	3 (11.54%)	16 (26.23%)	0.22
Skin lesions	30 (56.60%)	92 (61.33%)	0.66	14 (53.85%)	37 (60.66%)	0.72
Oral ulcer	12 (22.64%)	25 (16.67%)	0.45	7 (26.92%)	6 (9.84%)	0.05
Raynaud phenomenon	12 (22.64%)	27 (18.00%)	0.59	7 (26.92%)	11 (18.03%)	0.52
Cavities	10 (18.87%)	3 (2.00%)	0.00	3 (11.54%)	3 (4.92%)	0.36
Epistaxis	4 (7.55%)	8 (5.33%)	0.52	2 (7.69%)	3 (4.92%)	0.63
Arthritis	29 (54.72%)	78 (52.00%)	0.86	16 (61.54%)	44 (72.13%)	0.47

Proteinuria	26 (49.06%)	101 (67.33%)	0.03	15 (57.69%)	34 (55.74%)	1.00
Hematuria	26 (49.06%)	83 (55.33%)	0.53	12 (46.15%)	34 (55.74%)	0.56
Interstitial lung disease	4 (7.55%)	7 (4.67%)	0.48	2 (7.69%)	4 (6.56%)	1.00
Pulmonary arterial hypertension	8 (15.09%)	33 (22.00%)	0.38	8 (30.77%)	16 (26.23%)	0.86
Vasculitis	4 (7.55%)	14 (9.33%)	0.79	0 (0%)	3 (4.92%)	0.55
**Laboratory findings**						
Anemia	26 (49.06%)	79 (52.67%)	0.77	11 (42.31%)	35 (57.38%)	0.29
Leukocytopenia	19 (35.85%)	56 (37.33%)	0.98	13 (50.00%)	18 (29.51%)	0.11
Thrombocytopenia	12 (22.64%)	33 (22.00%)	1.00	8 (30.77%)	15 (24.59%)	0.74
Hypoalbuminemia	27 (50.94%)	82 (54.67%)	0.76	12 (46.15%)	31 (50.82%)	0.87
Increased blood urea nitrogen	8 (15.09%)	52 (34.67%)	0.01	2 (7.69%)	19 (31.15%)	0.04
Increased serum creatinine	4 (7.55%)	32 (21.33%)	0.04	1 (3.85%)	6 (9.84%)	0.67
Increased erythrocyte sedimentation rate	35 (66.04%)	96 (64.00%)	0.92	16 (61.54%)	47 (77.05%)	0.22
Increased C-reactive protein	19 (35.85%)	52 (34.67%)	1.00	10 (38.46%)	30 (49.18%)	0.49
Hypocomplementemia	36 (67.92%)	105 (70.00%)	0.91	21 (80.77%)	43 (70.49%)	0.47
ANA positive	28 (52.83%)	84 (56.00%)	0.81	16 (61.54%)	28 (45.90%)	0.27
Anti-dsDNA positive	29 (54.72%)	88 (58.67%)	0.74	16 (61.54%)	36 (59.02%)	1.00
Anti-SSA positive	44 (83.02%)	86 (57.33%)	0.00	22 (84.62%)	35 (57.38%)	0.03
Anti-Ro52 positive	36 (67.92%)	52 (34.67%)	0.00	14 (53.85%)	24 (39.34%)	0.31
Anti- SSB positive	24 (45.28%)	24 (16.00%)	0.00	10 (38.46%)	7 (11.48%)	0.01
Anti-Sm positive	17 (32.08%)	53 (35.33%)	0.79	6 (23.08%)	21 (34.43%)	0.43
Anti-RNP positive	12 (22.64%)	35 (23.33%)	1.00	5 (19.23%)	17 (27.87%)	0.56
Anti-cardiolipin positive	6/48 (12.50%)	10/103 (9.71%)	0.81	2/21 (9.52%)	4/45 (8.89%)	1.00

RF positive	19 (35.85%)	13 (8.66%)	0.00	6 (23.08%)	11 (18.03%)	0.80
IgG elevation	19 (35.85%)	33 (22.00%)	0.07	11 (42.31%)	20 (32.79%)	0.55

Table S3. Clinical characteristics of SS and SLE-SS in discovery and validation cohorts.

	**Discovery cohort**			**Validation cohort**		
**Characteristics**	**SLE-SS (N = 54)**	**SS (N = 133)**	**P value**	**SLE-SS (N = 25)**	**SS (N = 54)**	**P value**
**Demographic features**						
Female	52 (96.30%)	119 (89.47%)	0.16	23 (92.00%)	49 (90.74%)	1.00
Age at diagnosis (years)	36 [27.00-51.00]	52 [40.00-65.00]	0.00	45 [33.00-55.00]	49 [37.00-59.25]	0.38
Complicated with hypertension	8 (14.81%)	30 (22.56%)	0.32	7 (28.00%)	12 (22.22%)	0.78
Complicated with diabetes	1 (1.85%)	15 (11.28%)	0.04	2 (8.00%)	5 (9.26%)	1.00
**Clinical features**						
Fever	26 (48.15%)	19 (14.29%)	0.00	8 (32.00%)	10 (18.52%)	0.30
Alopecia	9 (16.67%)	8 (6.02%)	0.05	5 (20.00%)	5 (9.26%)	0.27
Asthenia	10 (18.52%)	33 (24.81%)	0.46	8 (32.00%)	3 (5.56%)	0.00
Dry mouth	34 (62.96%)	122 (91.73%)	0.00	18 (72.00%)	50 (92.59%)	0.03
Dry eye	26 (48.15%)	83 (62.41%)	0.10	9 (36.00%)	39 (72.22%)	0.01
Photosensitivity	14 (25.93%)	5 (3.76%)	0.00	4 (16.00%)	1 (1.85%)	0.03
Skin lesions	31 (57.41%)	8 (6.02%)	0.00	13 (52.00%)	7 (12.96%)	0.00
Oral ulcer	13 (24.07%)	14 (10.53%)	0.03	6 (24.00%)	6 (11.11%)	0.18
Raynaud phenomenon	13 (24.07%)	23 (17.29%)	0.39	6 (24.00%)	14 (25.93%)	1.00

Cavities	9 (16.67%)	26 (19.55%)	0.80	4 (16.00%)	10 (18.52%)	1.00
Epistaxis	4 (7.41%)	7 (5.26%)	0.73	2 (8.00%)	4 (7.41%)	1.00
Arthritis	30 (55.56%)	35 (26.32%)	0.00	15 (60.00%)	16 (29.63%)	0.02
Proteinuria	28 (51.85%)	34 (25.56%)	0.00	13 (52.00%)	12 (22.22%)	0.02
Hematuria	27 (50.00%)	26 (19.55%)	0.00	11 (44.00%)	11 (20.37%)	0.06
Interstitial lung disease	3 (5.56%)	23 (17.29%)	0.06	3 (12.00%)	10 (18.52%)	0.54
Pulmonary arterial hypertension	7 (12.96%)	22 (16.54%)	0.70	9 (36.00%)	9 (16.67%)	0.11
Vasculitis	4 (7.41%)	3 (2.26%)	0.11	0 (0.00%)	2 (3.70%)	1.00
**Laboratory findings**						
Anemia	28 (51.85%)	41 (30.83%)	0.01	9 (36.00%)	17 (31.48%)	0.89
Leukocytopenia	19 (35.19%)	44 (33.08%)	0.92	13 (52.00%)	18 (33.33%)	0.18
Thrombocytopenia	12 (22.22%)	28 (21.05%)	1.00	8 (32.00%)	10 (18.52%)	0.30
Hypoalbuminemia	28 (51.85%)	23 (17.29%)	0.00	11 (44.00%)	8 (14.81%)	0.01
Increased blood urea nitrogen	8 (14.81%)	10 (7.52%)	0.21	2 (8.00%)	3 (5.56%)	0.65
Increased serum creatinine	4 (7.41%)	6 (4.51%)	0.48	1 (4.00%)	0 (0%)	0.32
Increased erythrocyte sedimentation rate	36 (66.67%)	77 (57.89%)	0.34	15 (60.00%)	25 (46.30%)	0.37
Increased C-reactive protein	20 (37.04%)	34 (25.56%)	0.16	9 (36.00%)	14 (25.93%)	0.52
Hypocomplementemia	38 (70.37%)	93 (69.92%)	1.00	19 (76.00%)	33 (61.11%)	0.30
ANA positive	30 (55.56%)	57 (42.86%)	0.16	14 (56.00%)	22 (40.74%)	0.31
Anti-dsDNA positive	30 (55.56%)	14 (10.53%)	0.00	15 (60.00%)	8 (14.81%)	0.00
Anti-SSA positive	44 (81.48%)	93 (69.92%)	0.15	22 (88.00%)	38 (70.37%)	0.16
Anti-Ro52 positive	36 (66.67%)	72 (54.14%)	0.16	14 (56.00%)	30 (55.56%)	1.00

Anti- SSB positive	23 (42.59%)	41 (30.83%)	0.17	11 (44.00%)	21 (38.89%)	0.85
Anti-Sm positive	16 (29.63%)	4 (3.01%)	0.00	7 (28.00%)	6 (11.11%)	0.10
Anti-RNP positive	12 (22.22%)	12 (9.02%)	0.03	5 (20.00%)	11 (20.37%)	1.00
Anti-cardiolipin positive	6/93 (6.45%)	6/48 (12.50%)	0.34	2/21 (9.52%)	3/31 (9.68%)	1.00
RF positive	20 (37.04%)	41 (30.83%)	0.52	5 (20.00%)	23 (42.59%)	0.09
IgG elevation	19 (35.19%)	44 (33.08%)	0.92	11 (44.00%)	16 (29.63%)	0.32
